# Tips for erythropoiesis-stimulating agent treatment of renal anemia

**DOI:** 10.1007/s10157-019-01791-w

**Published:** 2019-10-16

**Authors:** Naohisa Tomosugi, Yoshitaka Koshino

**Affiliations:** 1 Medical Research Institute, Kanazawa Medical University, Ishikawa, Japan; 2 Mizuho Hospital, Ishikawa, Japan

**Keywords:** Renal anemia, Erythropoiesis-stimulating agent, Red blood cell, Mean corpuscular hemoglobin

*To The Editor,*

According to global guidelines on renal anemia, based on the best information on hemoglobin (Hb) levels for optimal prognosis, the recommended treatment strategy is to use an erythropoiesis-stimulating agent (ESA) and iron to maintain Hb levels at 10–12 g/dl [1–3]. On the basis of these guidelines, all clinicians vary the dose of ESA to adjust Hb levels, when they deviate from this range. However, red blood cell count (RBC), which is directly affected by ESA, is not addressed at all.

In patients with renal anemia, (1) erythroblast count is maintained by ESA, (2) each erythroblast synthesizes Hb in accordance with the supply of serum iron, (3) the Hb in matured RBCs transports oxygen to the mitochondria and adenosine triphosphate (ATP) is produced to generate energy. In the guidelines, this total Hb value is used as an index for treatment. In clinical practice, erythrocytes in a blood sample are lysed by sodium lauryl sulfate (SLS), used as a surfactant, which rapidly forms a complex with the released hemoglobin. As the complex of SLS-Hb has a characteristic spectrum with maximum absorbance at 539 nm, Hb value is calculated by measuring the absorbance. This method is based on the sum of Hb contained in each RBC (RBC-Hb), which is clinically impossible to measure. In other words, Hb value is essentially derived from two factors: RBC count and RBC-Hb. Mean corpuscular hemoglobin (MCH) was first introduced by Wintrobe in 1929 to define the hemoglobin content of RBCs [4]. He proposed the following formula to calculate MCH; MCH (pg/cell) = Hemoglobin (in g/1000 ml of blood)/RBC (in millions/ml).

**Fig. 1:**
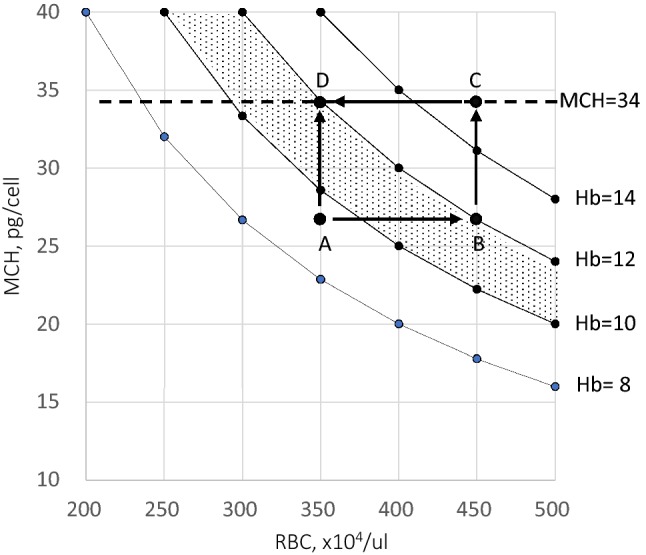
Advantage of the equation Hb = RBC × MCH with an upper limit against MCH in the treatment of renal anemia. Dotted line shows MCP = 34 pg/cell

When the value of Hb in the function MCH = Hb/RBC is fixed at 8, 10, 12, or 14 g/dl, RBC and MCH are shown as variables in the graph (Fig. 1). It shows that the condition to satisfy Hb 12 g/dl can be fulfilled by a variety of combinations of RBC and MCH. For example, when MCH remains 26.7 pg/cell and RBC count is increased from 350 × 10^4^/μl (Point A) to 450 × 10^4^/μl (Point B), Hb level increases from 9.3 g/dl to 12 g/dl. This value of Hb can also be achieved, when RBC remains at 350 × 10^4^/μl and MCH is increased from 26.7 pg/cell to 34.3 pg/cell (Point D). Since the guidelines recommend that Hb should be only maintained in the range of 10–12 g/dl (shadow on the graph), both Point B and Point D are estimated as good controls. The advantage of this equation, Hb = RBC × MCH is that there is an upper limit against MCH, which falls normally within the range 26–33 pg/cell [5]. MCH is decreased in iron deficiency (anemia) and increased in folic acid and vitamin B12 deficiencies that are observed in dialysis patients [6]. Assuming that MCH is below 34 pg/cell, Hb level cannot exceed 12 g/dl, when RBC count is maintained at 350 × 10^4^/μl (Point D), but Hb level can easily exceed 12 g/dl and rise as much as to 15.3 g/dl (Point C), when the RBC count is 450 × 10^4^/μl. In this case, following the guidelines, the dose of ESA should be reduced to adjust Hb to 12 g/dl. For example, when Point C shifts to Point D in a patient with 60 kg body weight, total RBC-Hb iron is reduced from 2400 to 1880 mg, as noted using the following formula: iron content of hemoglobin (mg) = Hb (g/dl) × 0.34 (%) × Whole Blood volume (ml)/10. The balance of 520 mg is converted to stored iron, causing the ferritin level to rise. Considering this perspective, the technical tips in the treatment of renal anemia is to first set RBC at around 350 × 10^4^/μl and then to increase MCH to 34 pg/cell by oral iron supplementation. These methods enable us to treat renal anemia without the fear that Hb will exceed 12 g/dl or that ferritin levels will rise. If iron is supplied when RBC count is maintained at a level greater than 350 × 10^4^/μl, Hb level may easily exceed 12 g/dl.

There are various types of ESA and iron supplementation methods available to adjust RBC and MCH individually. ESA controls the total number of red blood cells by acting directly on erythroblast lineages that express erythropoietin (EPO) receptors. In this reaction, the apoptosis of erythroblast precursors and neocytolysis of young erythrocytes due to ESA concentration have been implicated [7, 8]. When serum iron concentration increases due to iron supplementation, individual erythroblasts take up serum iron via transferrin receptor 1 (TfR1) expressed on the membrane surface and synthesize erythroblast-Hb from heme and globin [9], which is converted to RBC-Hb. The TfR1 expression is controlled by post-transcriptional regulation that is dependent on intracellular iron concentration. The iron efflux via ferroportin from macrophages and/or intestinal epithelial cells to the blood is regulated by hepcidin-25, which is secreted in response to serum iron loading and inflammation [10].

There is no description about RBC and MCH in the guidelines, but they should be paid greater attention to, because we have methods to control the amount of RBC and RBC-Hb individually. Currently, the recommended targets are to bring the RBC count to 350 × 10^4^/μl first and then bring MCH to 30 pg/cell. This may also help reduce the doses of ESA and iron.
